# Release of *Pleurotus ostreatus* Versatile-Peroxidase from Mn^2+^ Repression Enhances Anthropogenic and Natural Substrate Degradation

**DOI:** 10.1371/journal.pone.0052446

**Published:** 2012-12-21

**Authors:** Tomer M. Salame, Doriv Knop, Dana Levinson, Sameer J. Mabjeesh, Oded Yarden, Yitzhak Hadar

**Affiliations:** 1 Department of Plant Pathology and Microbiology, The Robert H. Smith Faculty of Agriculture, Food and Environment, The Hebrew University of Jerusalem, Jerusalem, Israel; 2 Department of Animal Sciences, The Robert H. Smith Faculty of Agriculture, Food and Environment, The Hebrew University of Jerusalem, Jerusalem, Israel; Queen Mary University of London, United Kingdom

## Abstract

The versatile-peroxidase (VP) encoded by *mnp4* is one of the nine members of the manganese-peroxidase (MnP) gene family that constitutes part of the ligninolytic system of the white-rot basidiomycete *Pleurotus ostreatus* (oyster mushroom). VP enzymes exhibit dual activity on a wide range of substrates. As Mn^2+^ supplement to *P. ostreatus* cultures results in enhanced degradation of recalcitrant compounds and lignin, we examined the effect of Mn^2+^ on the expression profile of the MnP gene family. In *P. ostreatus* (monokaryon PC9), *mnp4* was found to be the predominantly expressed *mnp* in Mn^2+^-deficient media, whereas strongly repressed (to approximately 1%) in Mn^2+^-supplemented media. Accordingly, *in-vitro* Mn^2+^-independent activity was found to be negligible. We tested whether release of *mnp4* from Mn^2+^ repression alters the activity of the ligninolytic system. A transformant over-expressing *mnp4* (designated OE*mnp4*) under the control of the *β-tubulin* promoter was produced. Now, despite the presence of Mn^2+^ in the medium, OE*mnp4* produced *mnp4* transcript as well as VP activity as early as 4 days after inoculation. The level of expression was constant throughout 10 days of incubation (about 0.4-fold relative to *β-tubulin*) and the activity was comparable to the typical activity of PC9 in Mn^2+^-deficient media. *In-vivo* decolorization of the azo dyes Orange II, Reactive Black 5, and Amaranth by OE*mnp4* preceded that of PC9. OE*mnp4* and PC9 were grown for 2 weeks under solid-state fermentation conditions on cotton stalks as a lignocellulosic substrate. [^14^C]-lignin mineralization, *in-vitro* dry matter digestibility, and neutral detergent fiber digestibility were found to be significantly higher (about 25%) in OE*mnp4*-fermented substrate, relative to PC9. We conclude that releasing Mn^2+^ suppression of VP4 by over-expression of the *mnp4* gene in *P. ostreatus* improved its ligninolytic functionality.

## Introduction

*Pleurotus ostreatus*, the oyster mushroom, is a commercially important edible ligninolytic white-rot filamentous basidiomycete and a good model for the study of mechanisms involved in lignin biodegradation [1]–[3]. Mn^2+^ supplements to *P. ostreatus* cultures have been shown to enhance degradation of anthropogenic aromatic compounds [4]–[5] and lignin [6]–[10]. Extracellular manganese peroxidases (MnPs) are considered to be key players in the ligninolytic system [4]–[18].

The MnP gene family (*mnp*s) of *P. ostreatus* is comprised of five Mn^2+^-dependent peroxidases (*mnp3*, *6*, *7*, *8* and *9*) and four versatile-peroxidases (*mnp1*, *2*, *4* and *5*; VPs), all having related gene and protein structure. Mn^2+^-dependent peroxidases (EC 1.11.1.13) exclusively oxidize Mn^2+^ to Mn^3+^ [4], [15]. Mn^2+^ is an obligatory co-substrate for these enzymes, as it is required to complete the catalytic cycle. VPs (EC 1.11.1.16) possess two catalytic sites, one for the direct oxidation of low- and high-redox-potential compounds, and the second for oxidation of Mn^2+^ in a preferred manner [4], [14], [15], [19], [20]. This dual activity mode of action enables VPs to modify a wide range of substrates, and makes them attractive potential catalysts for a variety of biotechnological applications [15], [20]. VPs have been isolated and thoroughly characterized in *Pleurotus* and *Bjerkandera*. The existence of VPs was also reported in *Panus*, *Trametes*, *Dichomitus* and *Spongipellis* [15], [17].

Gene-expression analyses of *P. ostreatus* cultures have revealed that its nine *mnp*s are transcribed in glucose-peptone medium (GP) and their expression is differentially affected by Mn^2+^ supplements. This resulted in drastic up-regulation (200-fold increase) of the predominantly expressed Mn^2+^-dependent peroxidase-encoding genes *mnp3* and *mnp9*, obtaining 0.2 level of expression relative to *β-tubulin*. In contrast, Mn^2+^ supplement resulted in drastic down-regulation (0.03-fold level of expression relative to *β-tubulin*) of the VP4-encoding gene *mnp4*, which is by far the predominantly expressed gene in Mn^2+^-deficient medium, exhibiting 2.5-fold level of expression relative to *β-tubulin*. These findings provided an explanation for the lower activity levels detected in Mn^2+^-containing cultures [4], [8]–[10]. Conclusive proof for the predominance of VP4 under Mn^2+^ deficiency was provided by inactivation of *mnp4* via gene replacement [18]. Similar trends have been reported for *Pleurotus eryngii* and *Trametes versicolor*. Martínez et al. [21] and Collins et al. [22] portrayed these phenomena as a “biological contradiction”: the preferred substrate for VPs activity, Mn^2+^, also reduces their expression.

In this report we investigated the ligninolytic functionality of a genetically engineered *P. ostreatus* strain in which *mnp4*, the gene encoding the predominant VP, was released from repression by using a constitutive promoter, resulting in over-expression of *mnp4* despite the presence of Mn^2+^ in the culture. Hence, in this strain VP4 is active under the conditions favoring aromatic compounds and lignin degradation. This trait may be harnessed for its use in various biotechnological applications, such as for bioremediation and pretreatment of lignocellulosic substrates to provide feedstocks for ruminants feed and the biofuels industry.

## Materials and Methods

### Fungal and Bacterial Strains and Growth Conditions

*Pleurotus ostreatus* monokaryon strain PC9 (Spanish Type Culture Collection accession number CECT20311), which is a protoclone derived by dedikaryotization of the commercial dikaryon strain N001 (Spanish Type Culture Collection accession number CECT20600), was used throughout this study [23]. Fungal strains were grown and maintained in YMG medium [1% w/v glucose, 1% w/v malt extract (Difco), 0.4% w/v yeast extract (Difco)] [24] or GP medium [2% w/v glucose, 0.5% w/v peptone (Difco), 0.2% yeast extract (Difco), 0.1% w/v K_2_HPO_4_, 0.05% w/v MgSO_4_·7H_2_O] containing 27 µM Mn^2+^ [4], [25]. When required, 1.5% (w/v) agar was added to the appropriate medium. Liquid cultures were maintained in stationary 100-ml Erlenmeyer flasks containing 10 ml media. Solid-state fermentation was conducted using cotton stalks as a substrate (obtained from a cotton field after defoliation and harvest, dried at 60°C and ground to pass a 2-mm-pore-size screen in a Wiley mill), moistened with 8 ml of deionized water in either the presence or absence of 73 µM Mn^2+^, in glass jars (125-ml, 60 mm diameter×68 mm height, Wheaton). Cultures were incubated at 28°C in the dark. The inoculum for all growth conditions was one disk (5 mm diameter) of mycelium obtained from the edge of a young colony grown on solid medium and positioned at the center of the Petri dish, flask or jar. The azo dyes Orange II, Reactive Black 5, Amaranth and fungicide carboxin (Sigma-Aldrich) were added to a final concentration of 100 mg/l and 2 mg/l (LD_50_ = 0.16 mg/l), respectively, as specified. *Escherichia coli* JM109 cells (Promega) were used for standard cloning procedures according to the manufacturer’s protocol. Culture biomass production was measured as dry weight (oven-dried to a constant weight at 65°C) in liquid GP culture containing Orange II. Mycelial linear growth rate was determined by measuring the position of the advancing mycelial front (leading hyphae) in solid GP culture containing Orange II.

### Nucleic Acid Manipulation and Analyses

Molecular manipulations were carried out on the basis of standard protocols as described by Sambrook et al. [26]. Genomic DNA was extracted with the DNeasy Plant Mini Kit (Qiagen) from culture biomass first ground under liquid nitrogen with mortar and pestle. Nucleic acid concentration and purity measurements were performed using a NanoDrop-2000 apparatus (Thermo Scientific). PCR was performed in an Eppendorf Mastercycler Gradient Thermocycler using Phusion High-Fidelity PCR Master Mix (Finnzymes), with the primers listed in Table 1. Isolation and purification of DNA fragments from agarose gel or PCR amplification were performed using the Wizard SV Gel and PCR Clean-Up System (Promega). Cloning into plasmids was performed using the pGEM-T Vector System II (Promega). Plasmid DNA was purified using the QIAprep Spin Miniprep Kit (Qiagen). DNA endonuclease restriction and ligation were performed using restriction enzymes and T4 DNA Ligase from Fermentas. Total RNA was extracted from culture biomass first ground under liquid nitrogen with mortar and pestle, then homogenized with QIA shredder spin columns (Qiagen) and purified from the lysate using the RNeasy Plus Mini Kit (Qiagen). cDNA was synthesized using the qScript cDNA Synthesis Kit (Quanta Biosciences) (Invitrogen). Gene-expression analyses were performed on an ABI StepOnePlus Real-Time PCR Sequence Detection System and software (Applied Biosystems), using Power SYBR Green PCR Master Mix (Applied Biosystems), with the primers listed in Table 1 and an annealing temperature of 63°C, according to the manufacturer’s default operating procedures. cDNA synthesis and real-time PCR can be inhibited by contaminants (e.g. plant polyphenols and polysaccharides) co-extracted with the RNA isolated from the cotton stalks after solid-state fermentation [10]. To eliminate bias resulting from the presence of possible inhibitors, serial dilutions of the isolated RNA were prepared and, using real-time PCR, confirmed to produce amplification plots accurately corresponding to the dilution factor, all within the recommended working limits of the system (i.e. an RNA concentration of approximately 0.1 µg for cDNA synthesis, and the targeted gene’s amplification threshold cycle (Ct) ranging between 24 and 27 during real-time PCR). DNA fragments, plasmid inserts and RT-PCR amplicons were fully sequenced at the Center for Genomic Technologies of The Hebrew University of Jerusalem.

**Table 1:** Oligonucleotides used in this study.

Primer designation	Sequence (5′→3′)
btubPF	CCGCGGCCGCGGATGCTGTTGGGAGGAAACTAAAT
mnp4F-btubPR	GGAGAGCGTCTTGAAAGACATTCTGCATGGAAAAGAAGTTAGTCG
btubPR-mnp4F	CGACTAACTTCTTTTCCATGCAGAATGTCTTTCAAGACGCTCTCC
btubTF-mnp4R	GGTGATATGGACAATCCAACGATTACGATCCAGGGCTGTAGGA
mnp4R-btubTF	TCCTACAGCCCTGGATCGTAATCGTTGGATTGTCCATATCACC
btubTR	GCATGCGCATGCAAGGGCACAAAATGACATGAA
R4	GCTGGTAGCGGTGGTTTTT
*Gene-expression*	see reference [4]

### Construction of the *mnp4* Over-expression Cassette

**Figure 1:**
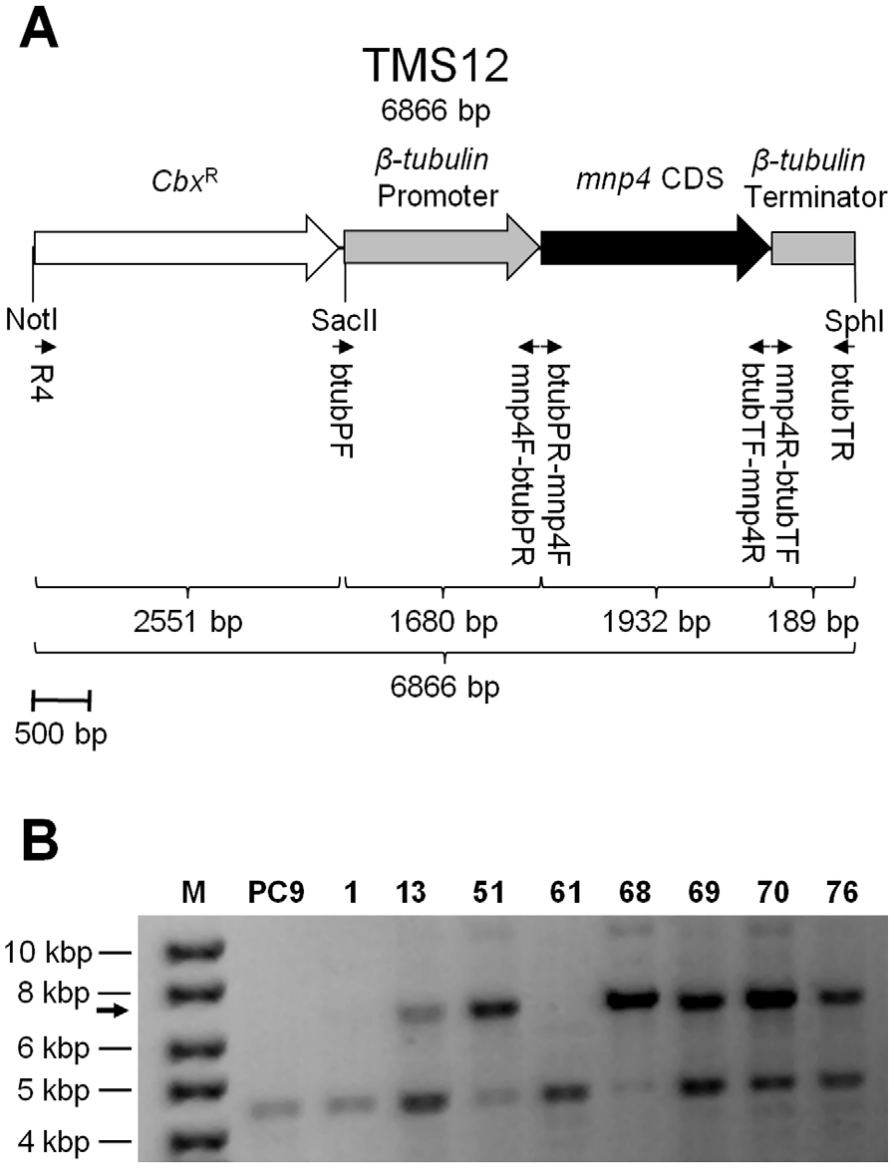
Strategy for producing a *mnp4* over-expressing *P. ostreatus* **strain.** (A) Map of the VP4 (encoded by *mnp4*) over-expression and carboxin-resistance-conferring (*Cbx* ^R^) cassette, TMS12. Small arrows indicate the location of primers (Table 1) used for construction and detection of the construct. (B) PCR screening of *P. ostreatus* genomic DNA targeting TMS12, using primers R4 and btubTR (Figure 1A, Table 1). The arrow indicates the expected 6866 bp amplicon. M – DNA size marker (GeneRuler DNA Ladder Mix, Fermentas); PC9– wild-type; 1, 13, 51, 61, 68, 69, 70, 76– carboxin-resistant transformant strains.

*β-tubulin* and *mnp4* were identified in the JGI genome database of PC9 v1.0 (http://genome.jgi-psf.org/PleosPC9_1/PleosPC9_1.home.html) as protein IDs 117235 and 137757, respectively, corresponding to previously identified protein IDs 16119 and 186006, respectively, in PC15 v1.0 [4], [15], [18]. The promoter and terminator regions of *β-tubulin* [8], [18] and the coding sequence (CDS) of *mnp4* were amplified from genomic DNA, using primers btubPF and mnp4F-btubPR, mnp4R-btubTF and btubTR, btubPR-mnp4F and btubTF-mnp4R, respectively (Figure 1A, Table 1). The resulting amplicons were fused together using the double-joint (fusion) PCR technique [27], [28] and then ligated into plasmid pTM1 [29] using the SphI and SacII restriction sites to produce a conjunct *mnp4* expression and carboxin-resistance (*Cbx* ^R^) cassette designated TMS12 (Figure 1A), which was cloned to produce plasmid pTMS12.

### Fungal Transformation

Transformation was performed based on the PEG-CaCl_2_ protocol previously adapted for *P. ostreatus*. Carboxin was used as a selection marker, and resistance was conferred via introduction of the carboxin-resistance cassette (*Cbx* ^R^) (Figure 1A). Either plasmid pTMS12 or the linear cassette TMS12, extracted from pTMS12 by restriction with NotI and SphI, were used as the transforming DNA (Figure 1A). Competent protoplasts were produced by digestion of vegetative mycelium of *P. ostreatus* from YMG liquid culture with lytic enzymes. The lytic enzyme solution consisted of 2% (w/v) Lysing enzymes from *Trichoderma harzianum* (Sigma-Aldrich, product number L1412) and 0.05% (w/v) Chitinase from *T. viride* (Sigma-Aldrich, product number C8241) in 0.5 M sucrose as an osmotic stabilizer. The protoplasts were washed (by centrifugation at 450 *g*, 8 min, 4°C) in STC solution (18.2% w/v sorbitol, 50 mM Tris-HCl pH 8.0, 50 mM CaCl_2_, 0.5 M sucrose), and adjusted to a final concentration of 5×10^7^ protoplasts/ml. Then, 2 ml protoplasts was mixed with 100 µl transforming DNA (300 ng/µl), 150 µl heparin solution (Sigma-Aldrich, product number H4784) (5 mg dissolved in 1 ml STC solution), and 300 µl single-strand λ phage carrier DNA (Fermentas, product number SD0011) (500 µg/ml, after denaturation at 95°C for 5 min and immediate transfer to ice). After 40 min of incubation on ice, 10 ml PTC solution (40% w/v PEG#4000, 50 mM Tris-HCl pH 8.0, 50 mM CaCl_2_, 0.5 M sucrose) was added, and the mixture was incubated for 20 min at room temperature. The mixture was then plated on selective solid YMG regeneration medium, containing 0.5 M sucrose and carboxin at a final concentration of 2 mg/l. Transformants were isolated after 10 days of incubation at 28°C. Transformant stability was verified by three successive transfers (inoculated from the edge of a 10-day-old colony) to solid medium without the selection drug, and then returning the transformant to solid culture conditions in which the selective drug was present.

### Dye Decolorization Analyses

Decolorization capacity was estimated in GP cultures supplemented with one of the tested compounds: Orange II (λ_max_ 483 nm), Reactive Black 5 (λ_max_ 597 nm) or Amaranth (λ_max_ 521 nm). In solid culture, decolorization capacity was estimated according to the visually decolourized area, as measured from the center of the inoculation point. In liquid culture, 100 µl of media were mixed with 900 µl phosphate buffered saline (0.1 M, pH 7.4), and the dye concentration in the media was quantified according to the absorption reading of the solution at its λ_max_, using a BioMate 3 spectrophotometer (Thermo Scientific), according to a standard curve. Non-inoculated media supplemented with the corresponding dye were used as a control. No decolorization was observed in non-inoculated media for any of the dyes [4].

### Elemental Analyses

Samples were either solid-state fermentation products after lyophilization and grinding to pass a 1-mm-pore-size screen in a Wiley mill or sections of fresh solid culture medium, excised using a scalpel. Metal element concentrations were determined by plasma emission spectrometry after acid digestion, via End-On-Plasma Inductively Coupled Plasma Atomic Emission Spectroscopy (ICP-AES) model ARCOS (Spectro GMBH), at The Z.B.M. Analytical Laboratory of The Hebrew University.

### Enzymatic Activity Assays

Samples of culture fluids were collected, centrifuged (4720 g, 10 min, 4°C) and maintained at 4°C. Enzymatic activity assays were conducted in a volume of 200 µl in microtiter plates at 32°C, using the Synergy 2 Multi-Mode Microplate Reader (BioTek). Mn^2+^-dependent and Mn^2+^-independent activities were determined using phenol red (Sigma-Aldrich) as the substrate. Oxidation of phenol red was measured by monitoring the *A*_610_ (ε = 22.0 mM^−1^ cm^−1^). The reaction mixture contained 0.1 mM MnSO_4_, 0.1 mM H_2_O_2_, 0.01% (w/v) phenol red, 25 mM lactate, 0.1% (w/v) bovine serum albumin and 20 mM sodium succinate buffer (pH 4.5). After incubation, the reaction was terminated by addition of NaOH to a final concentration of 80 mM. Activity in the absence of either MnSO_4_ or H_2_O_2_, was measured to establish specific (either Mn^2+^-dependent or Mn^2+^-independent) peroxidase activity. Accordingly, Mn^2+^-independent activity was deduced from the reaction in the absence of Mn^2+^ after subtraction of its corresponding reaction in the absence of H_2_O_2_, and Mn^2+^-dependent activity was deduced from the reaction containing Mn^2+^ by subtraction of the corresponding Mn^2+^-independent activity. Corresponding boiled samples served as blanks. One unit (U) of enzymatic activity was defined as the amount of enzyme that catalyzes the formation of 1.0 µmol of product per minute per milliliter of culture filtrate.

### Protein Expression Profile

Culture fluids were filtered through Whatman No. 1 filter paper followed by 0.45-µm mixed cellulose ester filter papers (Whatman). The sample was then concentrated 60-fold using a 10-kDa cutoff PM-10 membrane (Amicon Division) and treated with cOmplete, EDTA-free Protease Inhibitor Cocktail Tablets. The concentrated eluate was separated on a NuPAGE 4–12% bis-Tris gel in MES-SDS running buffer (Invitrogen), and the 40–45 kDa gel fraction was excised and maintained at 4°C. The sample was subsequently analyzed by HPLC/mass spectrometry/mass spectrometry (LC-MS/MS) in an Orbitrap (Thermo Scientific) mass spectrometer and identified by Sequest 3.31 software against the JGI genome database of *P. ostreatus* PC9 v1.0 (http://genome.jgi-psf.org/PleosPC9_1/PleosPC9_1.home.html), at The Smoler Proteomics Center of The Israel Institute of Technology (Technion).

### [^14^C]-lignin Mineralization Analysis

[**^14^**C]-lignin preparation and mineralization during solid-state fermentation were performed according to the methods described by Kerem and Hadar [6]–[8]. ^14^C-radiolabelled substrate (55×10^4^ dpm [^14^C]-lignin, 25±0.1 mg) was added to each of three replicates of 2 g of lignocellulosic substrate (cotton stalks) in 20-ml polyethylene cups (27 mm diameter×61 mm height), then sterilized, inoculated and sealed in a 300 ml biometer flask with two gas-tight caps. ^14^CO_2_ evolved in each flask was trapped every 3–4 days for 6 h on a cellulose filter paper (2×10 cm) presoaked with 0.25 ml of 6 N NaOH. Prior to removal of the filters, the flasks were flushed for 1 min with moistened, sterile, atmospheric air. Subsequently, the amount of trapped radioactive ^14^CO_2_ was measured in Hionic-Fluor (PerkinElmer) scintillation liquid by a TriCarb 1900 TR Liquid Scintillation Analyzer (Packard Instrument).

### Lignocellulose Digestibility Analyses

Non-inoculated substrate and solid-state fermentation products were lyophilized and ground to pass a 1-mm-pore-size screen in a Wiley mill. *In-vitro* dry matter digestibility (IVDMD) values were determined according to Tilley and Terry et al. [30], using rumen fluid obtained from two wether sheep (*Ovis aries*). Neutral detergent fiber digestibility (NDFD) values were determined by quantifying the NDF soluble content of samples pre-*in-vitro*-digestion and post-*in-vitro*-digestion according to the Van Soest methodology [31], adapted for the ANKOM-200 fiber analyzer (Ankom Technology). Acid detergent soluble compound, cellulose, acid-insoluble lignin and ash contents were determined according to the Van Soest methodology [31], adapted for the ANKOM-200 fiber analyzer (Ankom Technology).

### Statistical Analyses

Analysis of variance with Tukey-Kramer HSD test (significance accepted at *P*<0.05) was used to analyze differences among treatments, using JMP 7.0 Statistical Analysis Software (SAS Institute).

## Results

### Engineering a Strain Over-expressing *mnp4* (OE*mnp4*)

*P. ostreatus* VP4 is negatively regulated by Mn^2+^ in the culture medium [4], [8]–[10]. However, due to its potential importance and high ability to oxidize Mn^2+^ [14], [15], [18], [19], we produced a *P. ostreatus* strain over-expressing *mnp4* despite the presence of Mn^2+^ in the culture.

Introduced expression of *mnp2* [32] and *mnp3* [25], and knockdown (silencing) of *mnp3* by RNAi [4], [33] have been previously reported. In those studies, the transforming constructs were driven by the *P. ostreatus iron-sulfur protein subunit of succinate dehydrogenase* (*sdi1*) promoter. Recently, we have shown that the *P. ostreatus β-tubulin* promoter yields expression levels superior to those achieved with the *sdi1* promoter [18].

Therefore, we used the *β-tubulin* promoter to drive *mnp4* CDS in the TMS12 cassette (Figure 1A) transformed into the wild-type strain (PC9). About 100 carboxin-resistant colonies were isolated and, after confirming their stability, grown without antibiotic selection.

**Figure 2:**
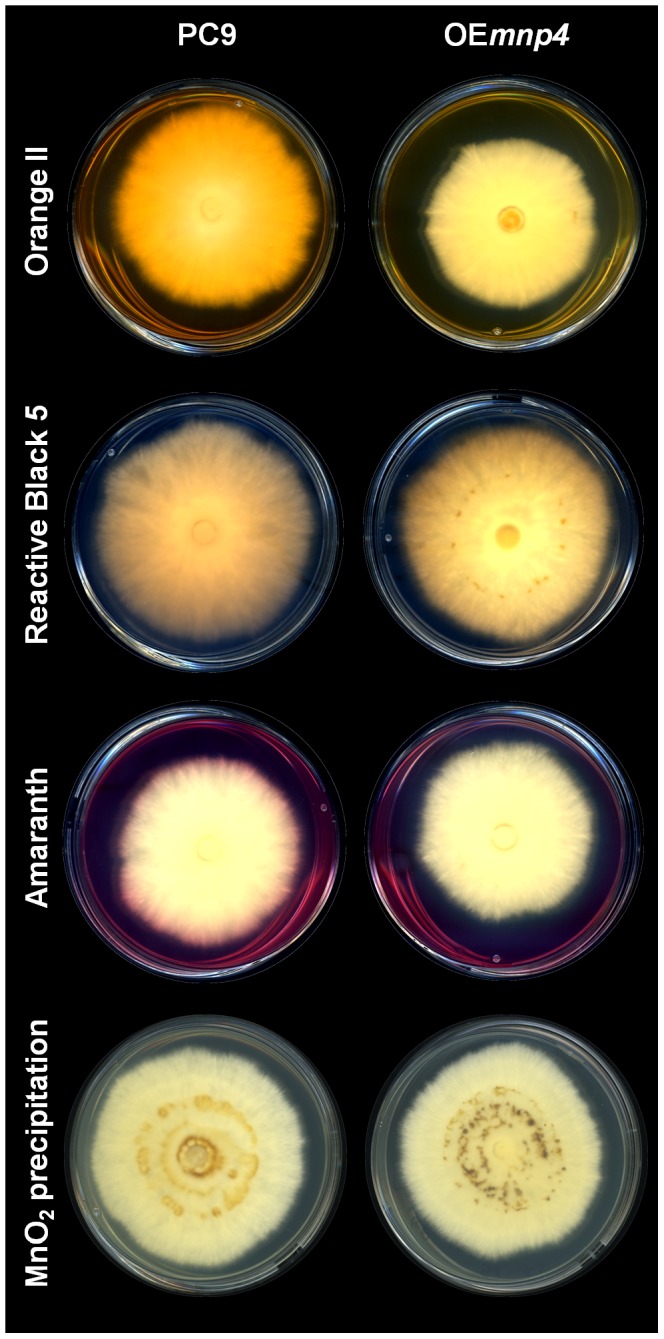
*In-vivo* decolorization of Orange II, Reactive Black 5 and Amaranth, and MnO_2_ precipitation. *P. ostreatus* wild-type strain (PC9) and *mnp4* over-expressing strain (OE*mnp4*) were grown for 10 days on solid GP medium containing 27 µM Mn^2+^ supplemented with 100 mg/l of the corresponding dye. For Mn^2+^ oxidation assay, the fungi were grown on solid GP medium containing 108 µM Mn^2+^.

Screening for candidate strains over-expressing *mnp4* was performed by evaluating Orange II decolorization in solid GP cultures [4]. More than 50% of the transformants exhibited markedly enhanced decolorization compared to PC9, while showing a similar linear growth rate. Similar results were observed using Reactive Black 5 and Amaranth (Figure 2) [4], Orange G, Bromocresol green, Poly R-478, Poly B-411 and Remazol Brilliant Blue R (data not shown).

An additional screen consisted of monitoring the appearance of dark brown to black precipitates typically visualized in solid GP cultures containing more than 54 µM Mn^2+^ (Figure 2). Elemental analyses confirmed that the precipitates formed in a culture containing 108 µM Mn^2+^ were composed of oxidized Mn^2+^, i.e. MnO_2_, as the dark areas contained 22.3±2.1 mg/kg Mn^2+^ in comparison to 5.0±1.4 mg/kg Mn^2+^ in the non-darkened areas. No differences were found in Mg^2+^ and K^+^ concentrations (internal controls) in these areas. An indication of enhanced Mn^2+^ oxidation by the transformants was the formation of larger and darker areas of MnO_2_ precipitates when grown in solid GP culture containing 108 µM Mn^2+^ (Figure 2).

Genomic integration of TMS12 was confirmed by PCR, using primers R4 and btubTR, on DNA extracted from eight transformants (designated 1, 13, 51, 61, 68, 69, 70 and 76) and PC9 (Figure 1, Table 1). Integration of the full-length TMS12 cassette was verified in strains 13, 51, 68, 69, 70 and 76 (Figure 1B). It is possible that although the TMS12 amplicon was not detected in strains 1 and 61, they did integrate sufficient essential segments from TMS12 to produce the phenotypes described above. A non-specific amplicon observed in all of the tested strains provided a convenient internal control, verifying the integrity of the DNA used (Figure 1B). Strain 51 was selected for further study, referred to herein as OE*mnp4*. Both PC9 and OE*mnp4* showed similar linear growth rates (7.3±0.2 and 6.8±0.2 mm/24 h, respectively) and biomass production (125.7±6.9 and 114.8±11.7 mg/flask, after 10 days of growth, respectively).

### Constitutive Over-expression of *mnp4* Results in Higher Peroxidase Activities and Earlier *in-vivo* Azo Dye Decolorization

**Figure 3:**
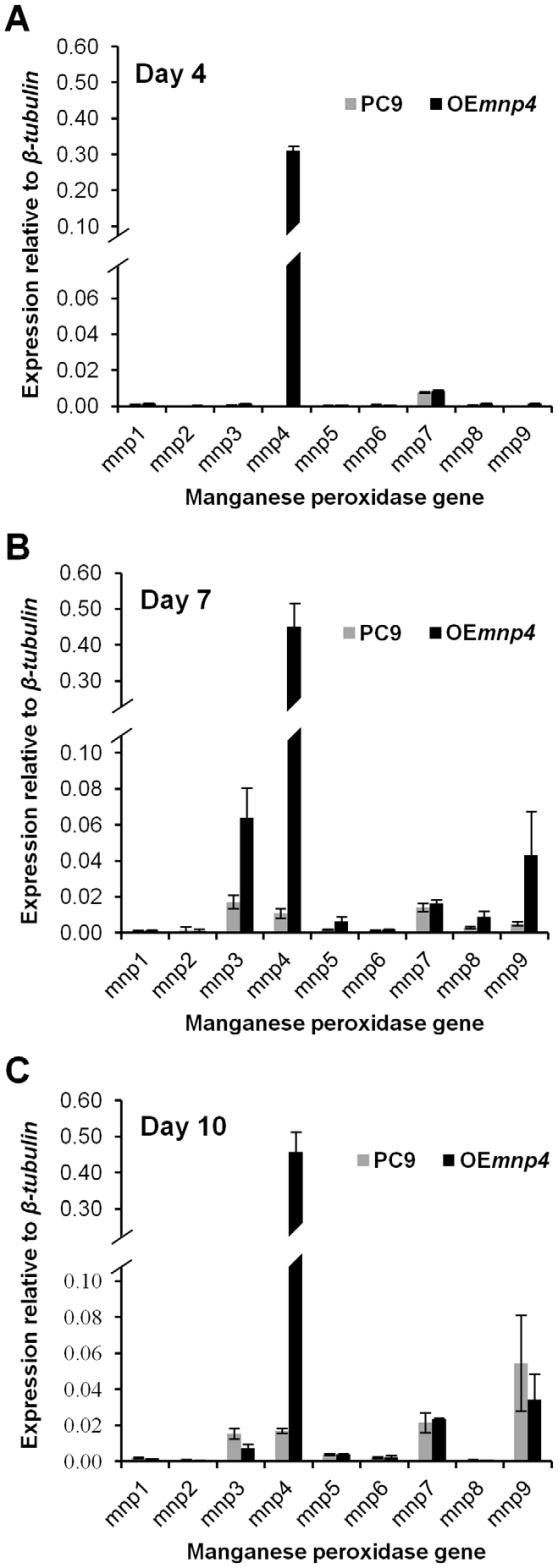
Expression of the *P. ostreatus manganese peroxidase* genes in PC9 and OE*mnp4* strains. Total RNA was extracted from *P. ostreatus* wild-type strain (PC9) and *mnp4* over-expressing strain (OE*mnp4*) cultures at (A) 4 days, (B) 7 days and (C) 10 days of incubation. The fungi were grown in GP medium containing 27 µM Mn^2+^ supplemented with 100 mg/l Orange II. Data represent the average of three biological replicates. Bars denote SD.

To determine whether OE*mnp4* over-expresses *mnp4* in culture despite the presence of Mn^2+^, the MnP gene family expression profiles of OE*mnp4* and PC9 were quantitatively evaluated in total RNA extracted from liquid GP cultures, at 4, 7 and 10 days of incubation (Figure 3).

Expression of the introduced *mnp4* was apparent at high levels as early as day 4, while the expression of the endogenous *mnp*s (in particular *mnp3*, *mnp4* and *mnp9*) was evident from day 7 onwards. *mnp4* expression increased throughout the incubation period (Figure 3).

To support these results, the 40–45 kDa fraction of the fungal secretome, which contains the peroxidases, also based on *in-silico* genomic analyses [14], [15], was analyzed for MnP isoenzymes in extracellular fluids collected from liquid GP culture at 7 days. 8, 6 and 4 unique peptides corresponding to VP4, MnP3 and MnP9, respectively, were detected, corresponding to the presence of significant amounts of *mnp4*, *mnp3*, and *mnp9* transcript.

Taken together, the genetic manipulation resulted in modification of three major traits with respect to *mnp4* expression: (a) release from Mn^2+^ repression, (b) markedly higher expression level, and (c) earlier expression. Specifically, OE*mnp4* expression of *mnp4* was at least 27-fold higher than that of PC9 throughout the incubation period.

**Figure 4:**
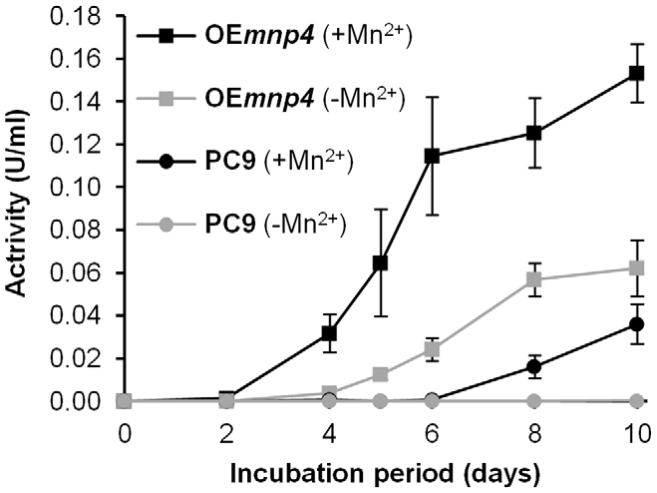
Time-course assay of peroxidase activity. Mn^2+^-dependent (+Mn^2+^) and Mn^2+^-independent (−Mn^2+^) peroxidase activities of *P. ostreatus* wild-type strain (PC9) and *mnp4* over-expressing strain (OE*mnp4*). The fungi were grown in GP medium containing 27 µM Mn^2+^ supplemented with 100 mg/l Orange II for 10 days. Data represent the average of three biological replicates. Bars denote SD.

To directly study the effect of *mnp4* over-expression on extracellular oxidation activity in the presence of Mn^2+^ in the culture, a 10-day time-course assay was conducted. Both of the VP activities, Mn^2+^-dependent and Mn^2+^-independent peroxidase-catalyzed reactions, were measured in liquid GP cultures of OE*mnp4* and PC9 (Figure 4). Phenol red was used as a substrate, and its oxidation was monitored in the presence and absence of Mn^2+^ in the reaction mixture.

In PC9, Mn^2+^-dependent activity was negligible until 8 days, reaching 0.016 U/ml, and gradually increased to a maximum 0.036 U/ml at 10 days. In OE*mnp4* it was detected 4 days earlier and was higher throughout the incubation period, reaching 0.032 U/ml at 4 days, and constantly increasing to 0.153 U/ml at 10 days, while still maintaining a rising trend (Figure 4). Thus, the level of Mn^2+^-dependent activity in OE*mnp4* was at least 4-fold higher than in PC9 throughout the incubation period.

As expected, owing to negative regulation of *mnp4* by Mn^2+^ [4], [8]–[10], Mn^2+^-independent activity in PC9 was negligible throughout the incubation period. In OE*mnp4*, the activity profile correlated with *mnp4* transcript levels, as higher activity was observed throughout the incubation period, reaching 0.004 U/ml at 4 days and constantly increasing to 0.062 U/ml at 10 days (Figure 4). This level of activity in OE*mnp4* was comparable to that observed in a Mn^2+^-deficient culture of PC9 [9], [18].

**Figure 5:**
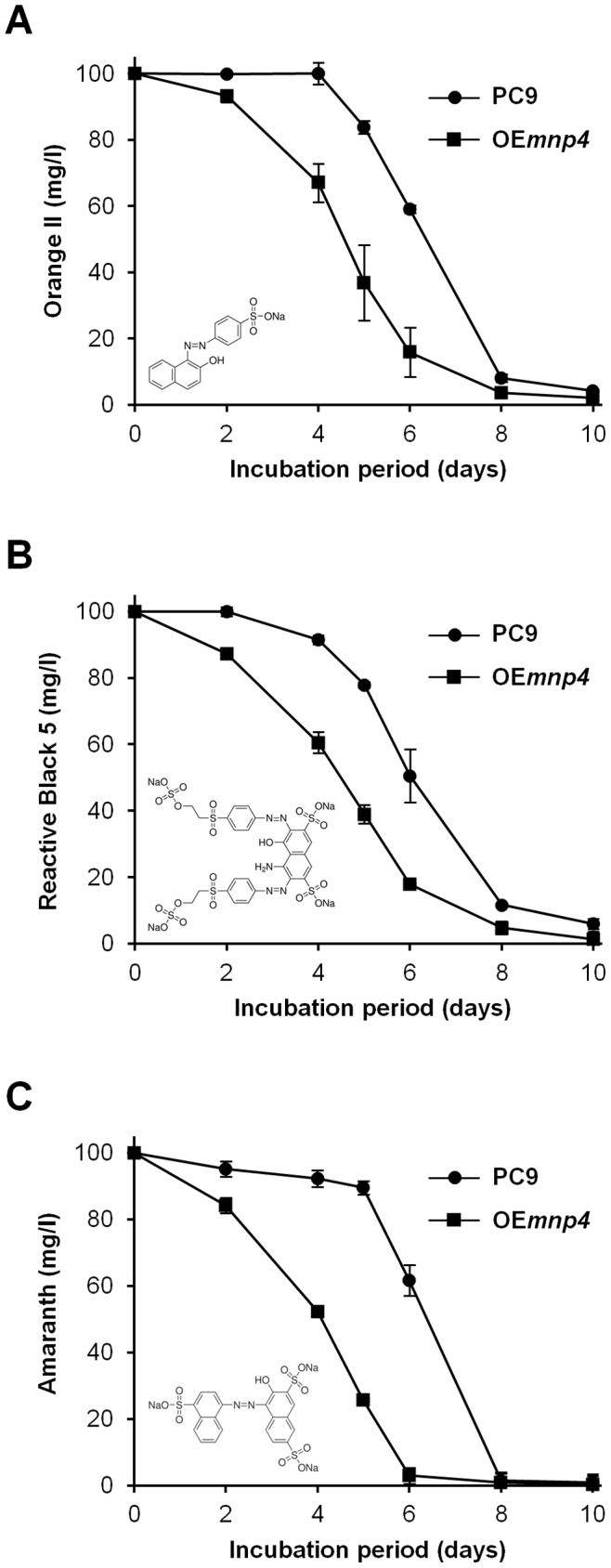
Time-course assay of *in-vivo* azo dyes decolorization. *P. ostreatus* wild-type strain (PC9) and *mnp4* over-expressing strain (OE*mnp4*) were grown for 10 days in GP medium containing 27 µM Mn^2+^ supplemented with 100 mg/l of (A) Orange II, (B) Reactive Black 5 and (C) Amaranth. Data represent the average of three biological replicates. Bars denote SD. The chemical structure of each dye is shown.

To evaluate the effect of *mnp4* over-expression on the function of the ligninolytic system, the *in-vivo* decolorization capacities of the azo dyes Orange II, Reactive Black 5 and Amaranth by OE*mnp4* and PC9 were determined in liquid GP cultures during 10 days of incubation (Figure 5).

The results clearly indicate earlier *in-vivo* azo dye decolorization capacity by OE*mnp4* (Figure 5). Furthermore, the earlier decolorization by OE*mnp4* correlated with both the high and early expression level of *mnp4* and the presence of Mn^2+^-mediated peroxidase activity at 4 days of incubation in OE*mnp4* culture, while at this time point the expression of the other *mnp*s and all the *mnp*s in PC9 were tenuous, as was the activity in PC9 culture (Figure 3, Figure 4).

In spite of the constant increased accumulation of VP activity in OE*mnp4* (Figure 4), the rate of decolorization of each of the tested dyes was comparable between OE*mnp4* and PC9 (Figure 5). This may suggest the existence of an as yet unknown rate-limiting factor restricting the expected accelerated decolorization by OE*mnp4* due to over-expression of VP4 (Figures 3 and 4).

### Enhanced [^14^C]-lignin Mineralization and Lignocellulose Digestibility by OE*mnp4*

**Figure 6:**
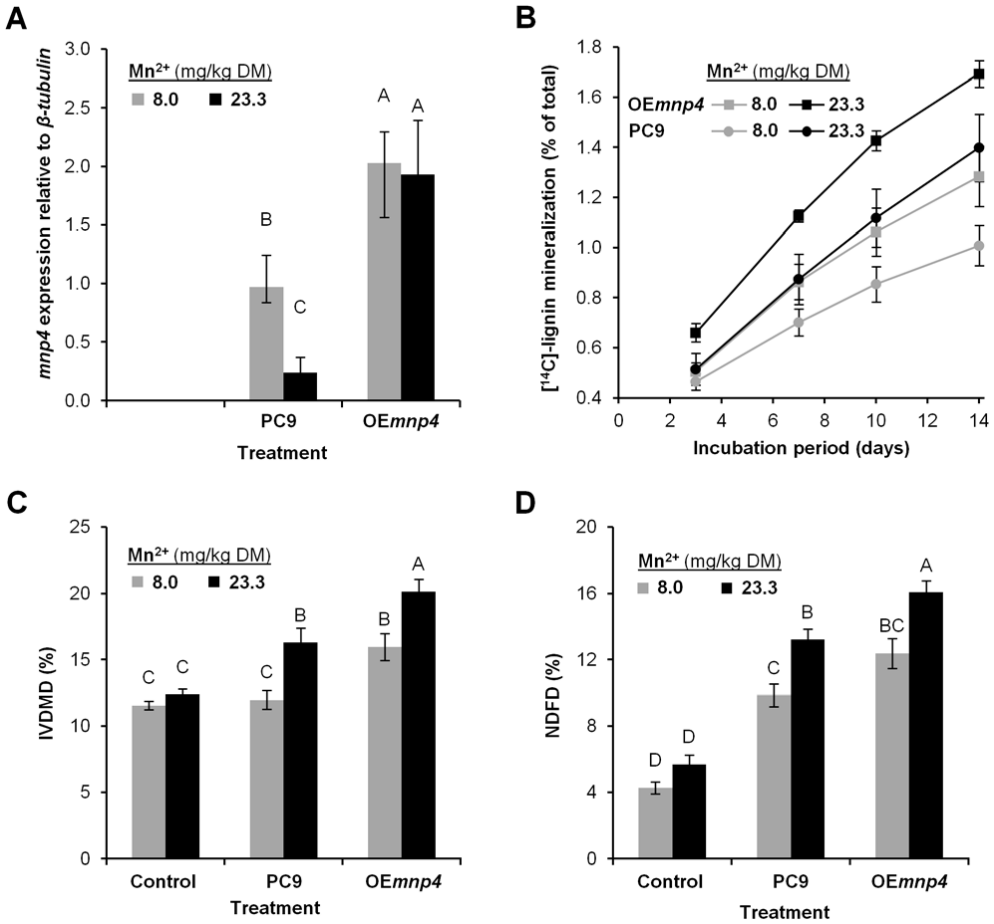
Gene-expression, lignin degradation and lignocellulose digestibility under solid-state fermentation. (A) *mnp4* expression level, (B) [^14^C]-lignin mineralization (percentage of ^14^CO2 emitted from the total initial radiolabelled [^14^C]-lignin is presented), (C) *in-vitro* dry matter digestibility (IVDMD) and (D) neutral detergent fiber digestibility (NDFD) by *P. ostreatus*. The wild-type strain (PC9) and *mnp4* over-expressing strain (OE*mnp4*) were incubated for 14 days under solid-state fermentation conditions, using a lignocellulosic substrate of cotton stalks containing either 8.0 (basal Mn^2+^ concentration) or 23.3 (concentration in Mn^2+^ supplemented stalks) mg/kg dry matter (DM) of Mn^2+^. Non-inoculated substrate was used as a control. Data represent the average of three biological replicates. Bars denote SD. Statistical analysis was performed by analysis of variance with Tukey-Kramer HSD test (significance accepted at *P*<0.05).

To assess the effect of *mnp4* over-expression on lignin degradation and the digestibility of lignocellulose, cotton stalks (non-supplemented or supplemented with Mn^2+^) were inoculated with OE*mnp4* or PC9 and incubated for 14 days under solid-state fermentation conditions. The cultures were then analyzed to quantify *mnp4* expression level in total RNA (Figure 6A), [^14^C]-lignin mineralization (Figure 6B), and the fermentation product was analyzed for *in-vitro* dry matter digestibility (IVDMD) (Figure 6C) [30] and neutral detergent fiber digestibility (NDFD) (Figure 6D) [31]. Non-inoculated substrate was used as a control.

Mn^2+^ concentrations in the cotton stalks were 8.0±1.4 mg/kg dry matter (basal Mn^2+^ concentration) and 23.3±2.1 mg/kg dry matter (after Mn^2+^ addition), which is consistent with 0.5 kg stalks treated with 2 liters of a 73 µM Mn^2+^ solution.

*mnp4* expression in PC9 grown on cotton stalks that were either non- supplemented or supplemented with Mn^2+^ was 0.97 and 0.24 relative to *β-tubulin*, respectively, while *mnp4* expression in OE*mnp4* grown under the same conditions was 2.03 and 1.93 relative to *β-tubulin*, respectively (Figure 6A). This clearly shows that Mn^2+^ supplements down-regulated *mnp4* expression in PC9 (being four-fold higher under non-supplemented conditions) whereas it did not have a significant effect in OE*mnp4*, demonstrating that the latter indeed expresses *mnp4* in a Mn^2+^-independent manner. Moreover, marked over-expression of *mnp4* was measured in OE*mnp4* compared to PC9 (two- and eight-fold higher in cotton stalks non-supplemented and supplemented with Mn^2+^, respectively).

This is in agreement with previously reported down-regulation of *mnp4* by Mn^2+^ in liquid GP cultures of PC9, albeit at a different level [4], [8]. While the expression of *mnp4* in GP not-supplemented with Mn^2+^ (containing less than 0.1 µM Mn^2+^) was seventy-fold higher than in the presence of 27 µM Mn^2+^ [4], [8], in cotton stalks not-supplemented with Mn^2+^ it was only four-fold higher than in cotton stalks in which Mn^2+^ (Figure 6A) was added. The fact that cotton stalks contain a significant basal level of Mn^2+^, likely enforcing down-regulation of *mnp4*, may explain the smaller difference observed between the two conditions using cotton stalks as a substrate.

[^14^C]-lignin mineralization of cotton stalks not-supplemented with Mn^2+^ was found to be 1.00% and 1.28% for PC9 and OE*mnp4*, respectively, after 2 weeks of solid-state fermentation. When the stalks were supplemented with Mn^2+^, mineralization increased in both strains, reaching 1.40% and 1.69% for PC9 and OE*mnp4*, respectively. The mineralization rate of OE*mnp4* surpassed that of PC9 throughout the incubation period in each of the culture conditions (Figure 6B).

Kerem et al. (6,7) and Cohen et al. (8) previously reported that Mn^2+^ supplements increase lignin degradation. As this phenomenon was shown, here, to also occur by Mn^2+^-independent over-expression of *mnp4*, direct evidence for the correlation between the level of *mnp4* expression and lignin degradation is now substantiated. Moreover, the fact the over-expression of *mnp4* enhanced lignin mineralization indicates that the basal level of *mnp4* expression limits the natural lignin degradation capacity by *P. ostreatus*.

IVDMD of non-inoculated cotton stalks (regardless of Mn^2+^ supplements) were 12%. This value was not altered in cotton stalks in which PC9 was cultured in the absence of Mn^2+^ supplements. However, when OE*mnp4* was cultured under the same conditions it increased to 16%. These values increased to 16% and more than 20% when PC9 or OE*mnp4*, respectively, were cultured in the presence of the Mn^2+^ supplements (Figure 6C). The NDFD values supported the IVDMD results, and in addition revealed a significant increase, from 6% to 10%, in the case of PC9 cultured on stalks not-supplemented with Mn^2+^, compared to the non-inoculated controls (Figure 6D).

From these results, we deduced that solid-state fermentation of cotton stalks with PC9 increases lignocellulose digestibility, and that Mn^2+^ supplements improve this process by more than 33%. Evidently, this process can be greatly enhanced by application of OE*mnp4*, producing digestibility values higher than that of PC9 by more than 33% (non-supplemented) and 25% (supplemented), respectively.

Moreover, these marked improvements in digestibility resulted from a relatively short-duration of solid-state fermentation (14 days), thus limiting consumption of the cellulose constituent (48.3–49.5% of the produce dry matter) by the fungus in all treatments.

Comparison of the gene-expression data (Figure 6A) with the mineralization (Figure 6B) and digestibility data (Figure 6C, D) of PC9 and OE*mnp4* leads to several fundamental conclusions: (a) the higher expression level of *mnp4* in OE*mnp4* correlates with higher mineralization and digestibility; (b) since OE*mnp4* shows similar *mnp4* expression in both Mn^2+^-supplemented and non-supplemented substrates, the higher mineralization and digestibility levels in the presence of Mn^2+^ supplement is most probably due to the production of greater amounts of reactive Mn^3+^ by VP4, as more of its substrate, Mn^2+^, becomes available for oxidation, and (c) in PC9, Mn^2+^ supplements down-regulate *mnp4* expression while at the same time increase mineralization and digestibility. These results strongly support the hypothesis that the amount of *mnp4* produced by PC9 is a limiting factor for its lignin-degrading capacity, as the higher *mnp4* expression by OE*mnp4* was proven to enhance lignocellulose digestibility.

## Discussion

Gene families of multiple ligninolytic isoenzymes encoded by various, and apparently redundant structurally-related genes, are common among ligninolytic fungi [17]. For example, *Phanerochaete chrysosporium*, *Ceriporiopsis subvermispora* and *Phlebia chrysocreas* have at least 5, 4 and 7 different genes encoding MnPs, respectively [12], [34], [35], and *P. ostreatus*, *Coprinopsis cinerea*, and *Laccaria bicolor* have at least 7, 17 and 11 different genes encoding laccases, respectively [36]–[38].

Differential gene-expression among the members of a gene family is also characteristic. Studies of the catalytic properties of various ligninolytic isoenzymes reflect distinct differences in both the culture conditions and substrate specificities associated with their transcription and kinetic constants [4], [39], [40]. For example, 7 isoforms of MnPs were isolated from *C. subvermispora* cultured in salt medium, whereas 4 isoenzymes were fractioned in extracts derived from wood chips. The requirement for Mn^2+^ by each of these MnPs varied on the basis of the nature of the aromatic substrate (vanillylacetone, *o*-dianisidine, *p*-dianisidine, guaiacol) added to the reaction mixture [41]. Expression of the *P. chrysosporium* MnP gene family is regulated by nitrogen levels, Mn^2+^, heat shock, agitation and other factors. While *mnps 1–3* are expressed under various culture conditions, *mnp4* and *mnp5* seem to be actively transcribed only when the fungus is grown on wood-containing soil samples, and wood pulp, respectively [34], [42]. Recently, a comprehensive systematic transcriptomic analysis showed that *Postia placenta* and *P. chrysosporium* gene-expression patterns, e.g. cytochrome P450s and extracellular glycoside hydrolases, are substantially influenced by the wood species used as a substrate [i.e. when grown on aspen (*Populus grandidentata*) or pine (*Pinus strobus*)] [39].

The fact the *P. ostreatus* has a large number of distinct genes encoding transcripts of various isoenzymes belonging to the MnP gene family points to the potential redundancy and/or diversity of these enzymes. It also may imply that complex and versatile strategies are employed by this fungus for the degradation of aromatic and recalcitrant compounds such as amorphic lignin and azo dyes. As the enzymatic systems for lignin degradation operate in concert for the successful degradation of lignin or recalcitrant anthropogenic aromatic compounds, regulation of the MnP gene family expression and corresponding enzymatic activity is also dependent on environmental conditions and nutrient availability [1], [4], [15].

Mn^2+^ is both an active mediator of MnP, and a regulator of MnP, laccase, and lignin peroxidase (LiP) expression in various ligninolytic fungi, usually exhibiting significantly higher MnP expression in Mn^2+^-supplemented compared to Mn^2+^-deficient media [6], [21], [34], [35], [43]. Accordingly, in the model white-rot fungus *P. chrysosporium*, significantly higher expression of MnP was obtained in Mn^2+^-supplemented cultures, whereas significantly higher expression of LiP was obtained in Mn^2+^-deficient cultures [34]. These phenomena can be explained by the fact that MnP catalyzes Mn^2+^ oxidation, whereas LiP oxidizes phenols and non-phenolic aromatics, hence the production of these enzymes alternates depending on the substrates present in the culture, to best fit the required mode of action.

While both of the predominant *P. ostreatus* MnPs, MnP3 and MnP9, comply with this dogma, the predominant VP4 does not [4], [9]. As mentioned above, an apparent biological contradiction exists: although VP efficiently oxidizes Mn^2+^, its expression is repressed by Mn^2+^. One proposed explanation is that VP is expressed during the later stages of lignin degradation, when much of the Mn^2+^ present in wood has been exhausted, and functions in the further oxidation of lignin subunits. Another possible explanation is that the Mn^2+^-independent activity of VP functions similarly to that of *P. chrysosporium* LiP, but this activity only occurs at relatively higher concentrations of phenolic substrates due to lower catalytic efficiency (*k*_cat_/*K*_m_) compared to Mn^2+^ as a substrate [21], [22]. Furthermore, since *P. ostreatus* does not possess LiP, but does possess LiP-like activity as part of VP (which may be regarded as a MnP-LiP hybrid enzyme), it might also retain an expression pattern similar to that of LiP. In short, VP repression by Mn^2+^ does not yet have a functional explanation and remains an enigma. This study focused on investigating this phenomenon by implementing a genetic manipulation that facilitates constitutive expression of *mnp4* and characterizing the corresponding phenotypes associated with ligninolytic functionality.

Introduced expression of *mnp4* CDS under the control of the *β-tubulin* promoter enabled circumventing the Mn^2+^ expression-repressive effect targeting the endogenous *mnp4*, thereby achieving an expression level that was one order of magnitude higher than naturally present in Mn^2+^-containing culture. Furthermore, a high level of *mnp4* expression was detectable days before significant levels of any other *mnp*s were detected. This expression level was on the same scale as that of endogenous *mnp4* in Mn^2+^-deficient culture, making *mnp4* (encoding VP4) the predominantly expressed member of the MnP gene family in Mn^2+^-containing culture, while under natural conditions this predominance is overtaken by *mnp3* and *mnp9* (both encoding MnPs) [4].

As a consequence of *mnp4* over-expression, the naturally negligible VP activity levels were markedly and constantly increased as the incubation period progressed, to levels comparable to those naturally present in Mn^2+^-deficient cultures [9], [18]. Moreover, these VP activities were detected 4 days earlier than the first indication of MnP activity. This enzymatic activity profile correlated with the aforementioned gene-expression profile.

*mnp4* over-expression in an environment where Mn^2+^ is present resulted in enhanced decolorization capacity of azo dyes, lignin mineralization, and conversion of lignocellulosic substrate into product with greater digestibility. Taken together, these data prove that the VP encoded by *mnp4* makes a key contribution to ligninolytic functionality in *P. ostreatus*.

The improved degradation of anthropogenic and natural substrates shown here by genetic modification of *P. ostreatus* may provide a basis for harnessing its qualities for various biotechnological processes, such as improving lignocellulose digestibility for use as animal feed or biofuel and for bioremediation.
